# Roxadustat-Induced Central Hypothyroidism Masked by Uremia at the Initiation of Hemodialysis: A Case Report

**DOI:** 10.7759/cureus.76499

**Published:** 2024-12-28

**Authors:** Mariko Hashimoto, Yoshikuni Nagayama, Ayana Ichikura-Iida, Takashi Inoue

**Affiliations:** 1 Nephrology, Yokohama Municipal Citizen's Hospital, Yokohama, JPN

**Keywords:** dialysis, end-stage renal disease, hif-phi, hypothyroidism, roxadustat, uremia

## Abstract

Hypoxia-inducible factor prolyl hydroxylase inhibitor (HIF-PHI) is a novel class of orally administered medications for renal anemia in patients with end-stage renal disease (ESRD). Roxadustat, a HIF-PHI, has a structure similar to that of triiodothyronine and may work as an agonist for thyroid hormone receptor-beta in the pituitary gland and/or hypothalamus. Therefore, roxadustat may cause central hypothyroidism due to suppressing thyroid-stimulating hormone (TSH) release in the pituitary gland and/or thyrotropin-releasing hormone release in the hypothalamus. On the other hand, some symptoms of uremia and hypothyroidism, such as fatigue, anorexia, or edema, are common. Therefore, hypothyroidism might be masked by uremia in patients with ESRD. We herein report the case of a 74-year-old man with roxadustat-induced central hypothyroidism masked by uremia at the initiation of hemodialysis. After stopping roxadustat medication without thyroid hormone replacement therapy, the patient’s TSH, free triiodothyronine, and free tetraiodothyronine levels returned to the normal range rapidly, and uremic-like symptoms such as anorexia and physical fatigue also gradually recovered. The number of ESRD patients undergoing treatment for renal anemia with roxadustat might increase in the future; hence, close attention to roxadustat-induced central hypothyroidism in ESRD patients with uremia at the initiation of hemodialysis is essential.

## Introduction

Hypoxia-inducible factor prolyl hydroxylase inhibitor (HIF-PHI), which induces intrinsic erythropoiesis and internal iron utilization, is a novel class of orally administered medications for renal anemia in patients with end-stage renal disease (ESRD) [1-4]. Roxadustat, a HIF-PHI first launched in Japan in 2019 ahead of the rest of the world, has structural similarity with triiodothyronine and may be a selective activating ligand for thyroid hormone receptor-beta in the pituitary gland and/or hypothalamus [5]. Therefore, roxadustat can lead to central hypothyroidism by inhibiting the release of thyroid-stimulating hormone (TSH) from the pituitary gland and/or thyrotropin-releasing hormone from the hypothalamus. A possible association between roxadustat and hypothyroidism was identified through an analysis of the pharmacovigilance database in Japan [6]. Although the prevalence of roxadustat-induced central hypothyroidism in patients with ESRD remains unclear, the cases have been reported in Japanese hemodialysis patients [7-11]. On the other hand, some symptoms of uremia and hypothyroidism, such as fatigue, anorexia, or edema, are common [12]. Therefore, hypothyroidism might be masked by uremia in patients with ESRD. We herein report the case of a 74-year-old man with roxadustat-induced central hypothyroidism masked by uremia at the initiation of hemodialysis.

## Case presentation

A 74-year-old man with ESRD due to nephrosclerosis was admitted to the nephrology department of our tertiary care hospital for evaluation of dyspnea. Three months before the admission, the patient was referred to our department for renal dysfunction (serum creatinine, 7.34 mg/dL). The patient had a history of hypertension for 20 years. There was no prior history of smoking or diabetes. The patient’s clinical findings on admission were as follows: blood pressure, 150/66 mmHg; pulse rate, 66 beats/minute; body temperature, 36.2 °C; percutaneous oxygen saturation at room, 99%; height, 160 cm; and weight, 58.7 kg. The patient had no remarkable physical findings other than lower-extremity edema. Laboratory findings on admission are shown in Table 1.

**Table 1:** Laboratory findings on admission. Ab, antibody; ACTH, adrenocorticotropic hormone; Ag, antigen; ALP, alkaline phosphatase; ALT, alanine aminotransferase; ANA, antinuclear antibody; ANCA, antineutrophil cytoplasmic antibody; AST, aspartate aminotransferase; BNP, brain natriuretic peptide; BUN, blood urea nitrogen; CK, creatine kinase; CMV, cytomegalovirus; Cre, creatinine; CRP, C-reactive protein; DHEA-S, dehydroepiandrosterone-sulfate; ds-DNA, double-strand DNA; EBV, Epstein-Barr virus; EBNA, EBV nuclear antigen; eGFR, estimated glomerular filtration rate; FT4, free tetraiodothyronine; FT3, free triiodothyronine; GBM, glomerular basement membrane; γ-GTP, gamma-glutamyl transpeptidase; GH, growth hormone; HbA1c, hemoglobin A1c; HBV, hepatitis B virus; HCV, hepatitis C virus; HIV, human immunodeficiency virus; IgA, immunoglobulin A; IgG, immunoglobulin G; IgM, immunoglobulin M; LDH, lactate dehydrogenase; MPO, myeloperoxidase; PCR, polymerase chain reaction; PRL, prolactin; PR3, proteinase 3; PTH, parathyroid hormone; RF, rheumatoid factor; RNP, ribonucleoprotein; RPR, rapid plasma reagin; SARS-CoV-2, severe acute respiratory syndrome-Coronavirus-2; sIL-2R, soluble interleukin-2 receptor; STS, serologic test for syphilis; T-Cho, total cholesterol; TG, triglyceride; TIBC, total iron-binding capacity; TPO, thyroid peroxidase; TSH, thyroid-stimulating hormone; UA, uric acid; VCA, viral capsid antigen; WBC, white blood cell

Parameter	Value (reference range)
Hematology	
WBC count, µL^-1^	4,840 (3,300-8,600)
Hemoglobin, g/dL	8.3 (13.7-16.8)
Platelet count, 10^4^/µL	22.3 (15.8-34.8)
Blood chemistry	
Cre, mg/dL	10.52 (0.65-1.0)
eGFR, mL/minute/1.73 m^2^	4.3
BUN, mg/dL	106.1 (8.0-20.0)
Total protein, g/dL	5.7 (6.6-8.1)
Albumin, g/dL	2.6 (4.1-5.1)
UA, mg/dL	5.4 (0.0-7.0)
Na, mEq/L	138 (138-145)
K, mEq/L	5.5 (3.6-4.8)
Cl, mEq/L	107 (101-108)
Ca, mg/dL	8.8 (8.8-10.1)
P, mg/dL	7.4 (2.7-4.6)
Mg, mg/dL	2.2 (1.8-2.3)
AST, U/L	17 (13-30)
ALT, U/L	10 (10-42)
LDH, U/L	179 (124-222)
ALP, U/L	78 (106-322)
γ-GTP, U/L	9 (13-64)
CK, U/L	62 (59-248)
T-Cho, mg/dL	89 (<220)
TG, mg/dL	40 (<150)
Glucose, mg/dL	104 (73-109)
HbA1c, %	5.4 (4.9-6.0)
CRP, mg/dL	12.3 (0.0-0.14)
Fe, µg/dL	17 (40-188)
TIBC, µg/dL	209 (165-436)
BNP, pg/mL	100.2 (<18.4)
Immunology	
IgG, mg/dL	1066 (861-1747)
IgA, mg/dL	351 (93-393)
IgM, mg/dL	78 (33-183)
ANA, titer	40
Anti-ds-DNA Ab	negative
Anti-RNP Ab	negative
MPO/PR3-ANCA	negative/negative
Anti-GBM Ab	negative
RF, U/mL	<5 (<15)
Cryoglobulin	negative
sIL-2R, U/mL	1400 (204-587)
Ferritin, ng/mL	533.8 (21.8-274)
Intact PTH	456 (10-65)
HBV surface Ag	Negative
HCV Ab	Negative
STS (RPR method)	Negative
Treponema pallidum Ab	Negative
HIV Ab/T-Spot assay	Negative/negative
Influenza A/B-PCR	Negative/negative
SARS-CoV-2 PCR	Negative
EBV VCA-IgG/IgM	Positive/negative
EBV EBNA-IgG	Positive
CMV-IgG/IgM	Positive/negative
Parvovirus B19 IgM	Negative
Endocrinology	
DHEA-S, ng/mL	52 (5-253)
Cortisol, µg/dL	11.9 (3.7-19.4)
ACTH, pg/mL	62.9 (7.2-63.3)
GH, ng/mL	1.25 (<2.47)
PRL, ng/mL	157 (4.29-13.69)
TSH, µIU/mL	0.1849 (0.61-4.23)
FT3, pg/mL	1.17 (1.68-3.67)
FT4, ng/dL	0.57 (0.7-1.48)
Anti-TPO Ab, IU/mL	4.3 (<3.3)
Anti-thyroglobulin Ab, IU/mL	<10.0 (<19.3)

**Figure 1:**
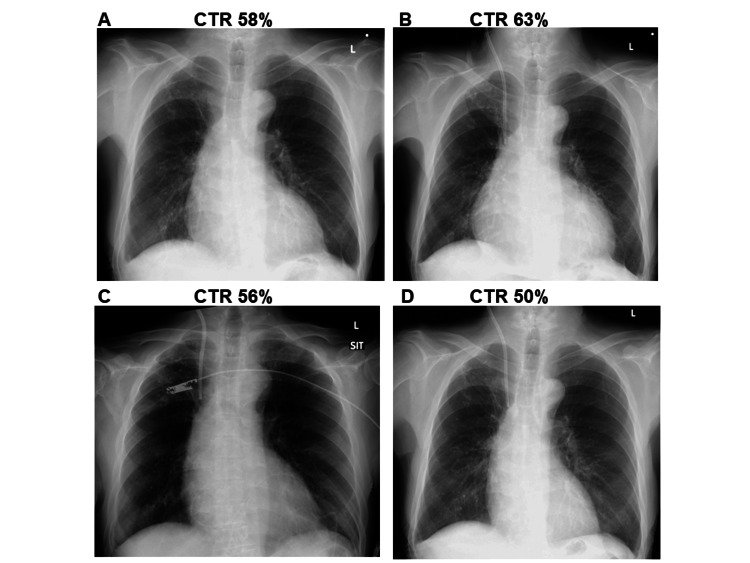
Changes in the cardiothoracic ratio (CTR) on chest radiography. (A) CTR 58% on admission; (B) CTR 63% on the 20th hospital day before pericardiocentesis; (C) CTR 56% on the 21st hospital day after pericardiocentesis; and (D) CTR 50% on the 30th hospital day.

Blood tests revealed anemia, hypoalbuminemia, renal dysfunction (serum creatinine, 10.52 mg/dL; estimated glomerular filtration rate [eGFR], 4.3 mL/minute/1.73 m^2^), elevated C-reactive protein, and elevated brain natriuretic peptide. The eGFR was calculated using the Japanese eGFR equation: (eGFR = 194 × serum creatinine^−1.094 ^× age^−0.287^) [13]. Serum ferritin and soluble interleukin-2 receptors were elevated. The patient lacked autoantibodies and infectious diseases, including hepatitis B virus, hepatitis C virus, syphilis, human immunodeficiency virus, tuberculosis, influenza, and severe acute respiratory syndrome Coronavirus-2. Investigation for Epstein-Barr virus, cytomegalovirus, and Parvovirus B19 showed a previous infection pattern. Blood cultures showed no microbes. Chest radiography revealed that the cardiothoracic ratio (CTR) was 58% without pleural effusion (Figure 1A).

**Figure 2:**
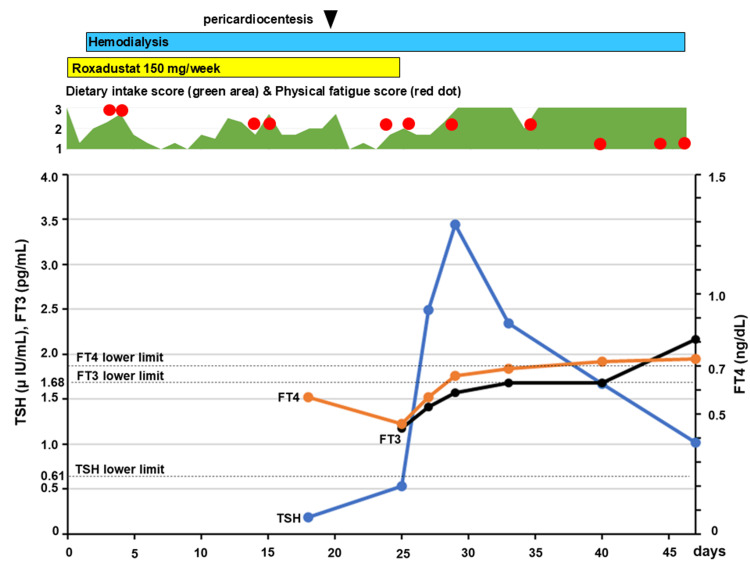
The clinical course of the patient. Changes in free tetraiodothyronine (FT4), free triiodothyronine (FT3), thyroid-stimulating hormone (TSH), dietary intake score, and physical fatigue score are shown. Dietary intake was assessed using a semi-quantitative score (full intake scored as 3, half intake as 2, and less than a small amount as 1), and physical fatigue was assessed using a semi-quantitative score of the patient’s feeling during a rehabilitation exercise therapy (being very tired scored as 3, being tired as 2, and being little tired to none as 1). The days on which the rehabilitation exercise therapy was conducted during hospitalization were only those indicated by red dots. After stopping roxadustat medication on the 26th hospital day, TSH, FT4, and FT3 levels returned to the normal range rapidly, and anorexia and physical fatigue also recovered gradually without thyroid hormone replacement.

There were no abnormalities on an electrocardiogram. Because of the aggravation of renal dysfunction and uremic symptoms such as anorexia and physical fatigue, hemodialysis was initiated on the second hospital day. The clinical course is illustrated in Figure 2. 

Although hemodialysis was started, the uremic symptoms did not improve, and the aggravation of pericardial effusion was observed. On chest radiography, the CTR was increased to 63% (Figure 1B). Echocardiography showed diffuse pericardial effusion; however, there was no right ventricular diastolic insufficiency, and left ventricular systolic function was normal. A gastroscopy finding was unremarkable. A computed tomographic (CT) scan of the chest and abdomen showed no significant malignant findings. On the 20th hospital day, a pericardiocentesis was performed to investigate the cause of the pericardial effusion. A 700 mL of hemorrhagic pericardial fluid was rapidly drained (Figure 1C). On cytologic examination, the pericardial fluid was classified as class II (no malignancy), corresponding to exudative according to Light’s criteria. Adenosine deaminase (ADA) level in the pericardial effusion was mildly elevated (42 U/L, reference range, 5.0-20.0); however, no significant causative microbes, including mycobacterium, were detected in cultures of both sputum and the pericardial fluid. T-Spot assay was negative. There was no re-accumulation of the pericardial fluid after the pericardiocentesis (Figure 1D); however, anorexia and physical fatigue continued. Brain CT showed no evidence of cerebrovascular disease or traumatic injury. Based on the endocrinological test, the patient’s symptoms were considered to be due to hypothyroidism (Table 1). Roxadustat (50 mg/day, three times per week) was administered orally for three months since the first referral; therefore, roxadustat-induced central hypothyroidism was suspected. After discontinuing roxadustat on the 26th hospital day, the TSH, free tetraiodothyronine (FT4), and free triiodothyronine (FT3) levels rapidly returned to the normal range without thyroid hormone replacement therapy, and clinical symptoms such as anorexia and physical fatigue gradually improved (Figure 2). Appetite and physical fatigue were assessed using a semi-quantitative evaluation of daily dietary intake (full intake scored as 3, half intake as 2, and less than a small amount as 1), and a semi-quantitative evaluation of the patient’s feeling during a rehabilitation exercise therapy (being very tired scored as 3, being tired as 2, and being little tired to none as 1), respectively. The days on which the rehabilitation exercise therapy was conducted during hospitalization were only those indicated by red dots (Figure 2). The patient was discharged without any significant symptoms on the 59th hospital day.

## Discussion

In this case, clinical symptoms, such as anorexia and physical fatigue, did not sufficiently improve after initiating hemodialysis or performing pericardiocentesis. However, these symptoms improved following the discontinuation of roxadustat, accompanied by the rapid increase of TSH, FT4, and FT3 levels, suggesting that roxadustat-induced central hypothyroidism was masked by uremia. It is known that non-thyroidal illnesses, such as starvation states and severe cachectic diseases, can also cause low T3 or low T4 syndrome. However, TSH levels are usually within the normal range in non-thyroidal illnesses [14]. In very serious cases, low TSH may also occur [15]. However, in this case, the patient was hospitalized in a general ward without undergoing any invasive major surgeries. Furthermore, symptoms suggesting hypothyroidism, such as bradykinesia, lethargy, and somnolence, also improved rapidly after the discontinuation of roxadustat. Therefore, the present patient was diagnosed with roxadustat-induced central hypothyroidism.

The pericardial effusion can occur due to various causes, including infections, malignancies, autoimmune diseases, and metabolic endocrine disorders such as uremia and myxedema [16]. Uremic pericarditis is currently considered to be rare (a prevalence of 1.7%) but one of the serious complications of ESRD at the initiation of renal replacement therapy [17]. In this case, the ADA level in the pericardial fluid was mildly elevated, but no mycobacterium was detected in cultures. Additionally, there was no re-accumulation of the pericardial fluid after drainage without anti-tuberculosis treatment, making a tuberculous etiology unlikely. Malignancy and autoimmune diseases were also ruled out, suggesting that the cause of the pericardial effusion was uremic pericarditis. Although there was a possibility that the pericardial effusion was associated with hypothyroidism, a challenge test involving the reintroduction of roxadustat was not performed, making it impossible to conclude.

Roxadustat-induced central hypothyroidism has been rarely reported in dialysis patients [7-11]; however, as seen in this patient, it can be masked by uremia at the initiation of hemodialysis. Therefore, it is important to monitor thyroid function when initiating roxadustat therapy, and careful attention to roxadustat-induced central hypothyroidism at the initiation of hemodialysis is also needed. The characteristics of patients prone to developing roxadustat-induced central hypothyroidism are unknown, and further accumulation of cases is needed.

## Conclusions

Some symptoms of uremia and hypothyroidism are common. Roxadustat, a HIF-PHI recently introduced for the treatment of renal anemia, may cause central hypothyroidism, which can be masked by uremia at the initiation of hemodialysis. Because the use of roxadustat for managing renal anemia in ESRD patients is expected to increase, it is important to monitor thyroid function when initiating therapy. Special attention should be given to roxadustat-induced central hypothyroidism in ESRD patients with uremia at the initiation of hemodialysis.
